# Investigating CD99 Expression in Leukemia Propagating Cells in Childhood T Cell Acute Lymphoblastic Leukemia

**DOI:** 10.1371/journal.pone.0165210

**Published:** 2016-10-20

**Authors:** Charlotte V. Cox, Paraskevi Diamanti, John P. Moppett, Allison Blair

**Affiliations:** 1 Bristol Institute for Transfusion Sciences, NHS Blood and Transplant, Filton, Bristol, United Kingdom; 2 School of Cellular and Molecular Medicine, University of Bristol, Bristol, United Kingdom; 3 Bristol Royal Hospital for Children, Bristol, United Kingdom; B.C. Cancer Agency, CANADA

## Abstract

A significant number of children with T-lineage acute lymphoblastic leukemia (T-ALL) fail to respond to therapy and experience early relapse. CD99 has been shown to be overexpressed on T-ALL cells and is considered to be a reliable detector of the disease. However, the relevance of CD99 overexpression in ALL has not been investigated in a functional context. The aim of this study was to investigate the functional capacity of CD99^+^ cells in childhood ALL and determine the suitability of CD99 as a therapeutic target. Flow cytometric analyses confirmed higher expression of CD99 in ALL blasts (81.5±22.7%) compared to normal hemopoietic stem cells (27.5±21.9%) and T cells (3.1±5.2%, P≤0.004). When ALL cells were sorted and assessed in functional assays, all 4 subpopulations (CD34^+^/CD99^+^, CD34^+^/CD99^-^, CD34^-^/CD99^+^ and CD34^-^/CD99^-^) could proliferate in vitro and establish leukemia in NSG mice. Leukemia propagating cell frequencies ranged from 1 in 300 to 1 in 7.4x10^4^ but were highest in the CD34^+^/CD99^-^ subpopulation. In addition, all four subpopulations had self-renewal ability in secondary NSG mice. Cells in each subpopulation contained patient specific TCR rearrangements and karyotypic changes that were preserved with passage through serial NSG transplants. Despite high levels of CD99 antigen on the majority of blast cells, leukemia initiating capacity in vivo was not restricted to cells that express this protein. Consequently, targeting CD99 alone would not eliminate all T-ALL cells with the ability to maintain the disease. The challenge remains to develop therapeutic strategies that can eliminate all leukemia cells with self-renewal capacity in vivo.

## Introduction

T-cell acute lymphoblastic leukemia (T-ALL) is a genetically heterogeneous cancer, with 20% of childhood patients and the majority of adult patients dying from resistant or relapsed disease.[1] Leukemia propagating cells (LPC) have been a topic of much investigation in recent years; they can self-renew to maintain the disease and some are resistant to current chemotherapeutic agents,[2–6] so these cells have the potential to cause relapse. LPC identified using immune deficient mouse models are diverse in terms of expression of CD34, CD1a, CD4 and CD7 antigens in both pediatric and adult T-ALL.[2–6] Since none of these markers are specific for LPC, distinguishing them from normal cells can be difficult.

Minimal residual disease (MRD) detection is a well-established tracking tool to assess therapeutic response. MRD levels during remission induction have become the most important prognostic indicator in ALL.[7–12] Consequently, they have been incorporated into global treatment protocols for risk assignment and selection of appropriate therapy (e.g. UKALL 2011, AIEOP-BFM ALL 2009, TOT XVI). In recent years, flow cytometric (FCM) panels have been developed to identify residual leukemia in a faster and more cost effective manner with good concordance (75–95%) with PCR-based analyses.[13, 14] EuroFlow antibody panels for standardized T-ALL diagnosis and MRD assessment[15] aim to discriminate T-ALL blasts from normal hemopoietic cells. CD99, a 32-kD transmembrane protein, is a leukemia associated phenotype marker used for the positive diagnosis of T-ALL. It is used with nuclear TDT, CD5, CD10 and CD1a as a panel that can discriminate T-ALL cells from normal hemopoietic cells.[15] Surface CD99 expression is high on pediatric T-ALL cells[16, 17] and absent on normal T cells. However, unlike some of the other markers in this panel, the relevance of CD99 overexpression has not been investigated in a functional context. Consequently, its role in leukemogenesis and potential as a therapeutic target are not known. Over-expression of cell surface antigens on leukemia cells has been exploited for therapeutic purposes e.g. Blinatumomab[18, 19] and CAR-T cell antigens against CD19 in B cell precursor (BCP) ALL.[20–22] Unfortunately, there is a lack of such targeted therapies for T-ALL. In this study we have investigated the expression of CD99 on LPCs that we have previously shown to be CD34^+^/CD7^+^, CD34^+^/CD7^-^ or CD34^-^ [5, 6] and assessed the functional capacity of cells sorted on the basis of surface CD99 expression in vitro and in vivo.

## Materials and Methods

### Sample sources

BM cells from children (median age, 6 years; range, 2–17 years) with T-ALL at presentation or relapse were obtained with written consent from parents or guardians and with approval of University Hospitals Bristol NHS Trust and the National Research Ethics Committee London-Brent. Detailed characteristics of the patient samples are shown in Table 1, below, and Table A in S1 File. Samples were selected on the basis of availability of material for study only. Normal BM (NBM) and peripheral blood (PB) samples were obtained from consented healthy donors. Cells were separated using Ficoll-Hypaque (Sigma-Aldrich, Poole, UK), mononuclear cells (MNC) were suspended in Iscoves modified Dulbeccos medium (IMDM; Invitrogen, Paisley, UK) with 90% fetal calf serum (FCS; Invitrogen) and 10% dimethyl sulfoxide (DMSO; Manor Park Pharmaceuticals, Bristol, UK) and stored in liquid nitrogen. Mean viability of samples on thawing was 83±17% for ALL samples and 71±17% for normal samples.

**Table 1:** Patient sample characteristics.

						% Nucleated cells			
ID	Karyotype	Age (y)	Sex	Disease status	MRD risk status	CD34^+^/CD99^+^	CD34^+^/CD99^-^	CD34^-^/CD99^+^	CD34^-^/CD99^-^
1	iso 9, +4	15	M	Relapse	Risk	1.70	0.80	95.00	2.50
2	t(11;14)	2	M	Relapse	Low	1.19	0.22	91.45	7.14
3	t(9;16),+7,-9	5	M	Relapse	Risk	2.70	1.80	78.90	16.60
4	46 XY	17	M	Diagnosis	Risk	58.10	1.34	29.30	11.26
5	t(1;14)	10	M	Diagnosis	Low	0.42	0.02	96.28	3.28
6	del 6q	15	M	Diagnosis	Risk	90.60	0.81	3.97	4.62
7	+1, del 6, del11	2	F	Diagnosis	N/A	0.09	0.01	64.7	35.2
8	46 XY	3	M	Diagnosis	Risk	0.11	0.07	87.5	12.32
9	complex	6	M	Diagnosis	Low	0.01	0.01	90.7	9.28

N/A, not available.

### Flow cytometry

Thawed human BM cells were labelled with the following antibodies from BD Biosciences (Oxford, UK), unless otherwise specified; anti-CD34 (clone 8G12)-APC and anti-CD38 (clone HB7)-PE or anti-CD3 (clone BW264/56)-FITC (Miltenyi Biotec, Surrey UK) and 7AAD (Sigma-Aldrich, Poole UK). Thawed MNC from 9 primary T-ALL cases were stained with anti CD34-APC, anti-CD7 (clone M-T701)-FITC, anti-CD99 (clone TÜ12)-PE and 7AAD. Cells were analyzed and sorted using a Becton Dickinson Influx cell sorter and BD Sortware version 1 (BD Biosciences), as outlined in Fig A in S1 File. Cells were initially gated on low forward and side scatter, excluding doublets and fluorescence minus one controls were used to set sort gates.

### Microarray analyses

Gene expression analysis of 5 normal BM and 5 T-ALL primary samples was performed using Agilent Whole Human Genome Oligo Microarrays (Miltenyi Biotec, Germany). Normal CD34^+^/CD38^-^ hemopoietic stem cells (HSC) and CD34^+^/CD7^+^, CD34^+^/CD7^-^, CD34^-^/CD7^+^, CD34^-^/CD7^-^ T-ALL subpopulations were sorted for whole genome array (WGA) analysis. Unsorted BM MNC and bulk T-ALL cells from the same respective sample cohorts were also used for WGA analyses. Cells were lyzed using SuperAmp Lysis Buffer according to the manufacturer’s instructions (Miltenyi Biotec). RNA amplification, cDNA preparation and hybridization were performed by Miltenyi Biotec as was subsequent gene-expression analysis using Agilent Whole Human Genome Oligo Microarrays. Fluorescence signals were detected using Agilent’s Microarray Scanner System (Agilent Technologies) and analyzed with the Agilent Feature Extraction Software. The entire data set of signal intensities was normalized by dividing the intensity value of each sample by the overall median, transformed to log scale and median centred. Differential gene expression analysis was performed on comparisons between T-ALL LPC versus normal HSC and bulk T-ALL versus bulk normal BM. Microarray data are available in the ArrayExpress database (www.ebi.ac.uk/arrayexpress) under accession number E-MTAB-4006.

### Suspension culture assay

Suspension cultures were initiated with unsorted ALL cells or with sorted subfractions at up to 5x10^5^ cells/mL, in IMDM supplemented with interleukin (IL)-3 (20ng/ml), IL-7 (10ng/ml) and stem cell factor (50ng/ml, all R&D systems, Abingdon UK). Cultures were maintained for up to 6 weeks with weekly half media changes. Cells removed during the media changes were used to determine viability and absolute counts by flow cytometry and for cytogenetic analyses by FISH, as described previously.[2]

### In vivo studies

In vivo experiments were carried out in accordance with the Animals (Scientific Procedures) Act under licenses granted by the United Kingdom Home Office (PPL 30/3113) and all efforts were made to minimize suffering, including killing via a schedule 1 method at the first sign of disease symptoms. NSG mice were bred and maintained at the University of Bristol Animal Service Unit. Mice were not preconditioned prior to inoculation. Cells were resuspended in 0.3mL IMDM + 5% human serum albumin and injected into the lateral tail veins of 6–8 week old mice. Unsorted cells and sorted cell populations were inoculated at a range of doses. Animals were monitored weekly for the presence of human cells in PB aspirates, maintained for up to 20 weeks and killed electively or as soon as they began to exhibit clinical symptoms of disease. The gross anatomy was inspected and femoral BM samples were removed for FCM and cytogenetic/histologic analyses. FCM analyses of PB and BM from xenografts were undertaken using antibodies against human CD7, CD19, CD34, CD45, CD99 and murine CD45 (all BD Biosciences). Aliquots of cells from NSG BM were plated onto slides for cytogenetic or morphological analysis.

Cells harvested from BM of some engrafted mice were used for serial transplantation experiments. These cells were not enriched for any particular phenotype before evaluation in sequential xenografts. Details of the number of cells inoculated into secondary recipients are provided in Table C in S1 File.

### Identification of clonal receptor gene rearrangements

Sorted ALL subfractions and cells recovered from BM of engrafted NSG mice were screened for the presence of T-cell receptor (TCR) rearrangements (TCRG, TCRD, and TCRB). DNA was extracted using QIAamp DNA Micro kit (QIAGEN, Crawley, United Kingdom). The polymerase chain reaction (PCR) primers and conditions used were as described previously.[2] PCR products were subjected to heteroduplex analysis, cloned and the monoclonal products were sequenced. Each rearrangement was characterized by comparison of sequences with germline sequences contained in online database www.imgt.org/IMGT_vquest/share/textes/.

### Cytogenetic analyses

Cytogenetic analysis by fluorescence in situ hybridization (FISH) was performed on samples at diagnosis, on cells harvested from cultures and cells harvested from NSG marrow by Bristol Genetics Laboratory, Southmead Hospital. At least 50 nuclei per sample were scored on preparations from cultured cells and those harvested from NSG mice.

### Statistical analyses

Analysis of variance (ANOVA) followed by Tukey’s post-hoc testing was used to compare CD99 antigen expression in the different cell sources and the proliferation of subpopulations in suspension culture. ANOVA and Tukey post-hoc tests were also used to identify significant differences in gene expression between groups of samples. Fold-change in gene expression between 2 samples was equivalent to their log2 intensity ratio. Bulk T-ALL gene expression was directly compared to BM MNC and that of each LPC subpopulation was compared to HSC. LPC frequencies were determined by Poisson statistics using L-Calc software (StemCell Technologies Inc). Matched paired T-tests were used to compare engraftment levels between primary and secondary NSG recipients.

## Results

**Fig 1:**
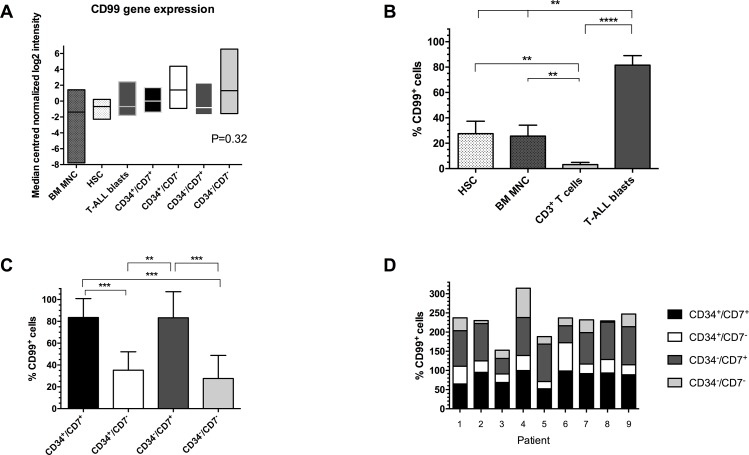
Expression of CD99 in T-ALL and normal hemopoietic cells. (A) Expression of CD99 antigen was assessed by flow cytometry in BM samples from 9 T-ALL cases and BM MNC, CD34^+^/CD38^-^ HSC and CD3^+^ T cells from 5–9 healthy donors. Lines represent median values, ** *P*≤0.007, **** *P*<0.0001. (B) Expression of CD99 in sorted LPC subpopulations from this T-ALL cohort. Lines represent median values, * *P* = 0.02, ** *P*≤0.009. (C) Stacked bar chart showing expression of CD99 in CD34/CD7 LPC subpopulations in individual cases. (D) Gene expression in BM samples from 5 T-ALL cases (pts 2, 4, 7, 8, 9) and from 5 healthy donors were analyzed using Agilent Whole Genome Oligo microarrays. Data shows side by side comparisons of median centered normalized log2 signal intensities. Boxes represent the range of expression and the horizontal lines represent the median.

BM samples from 9 children diagnosed with T-ALL and normal healthy donors were studied (Table 1). Initially CD99 expression, as determined by flow cytometry, was compared in normal BM MNC, CD34^+^/CD38^-^ HSC, CD3^+^ T cells and T-ALL blasts (Fig 1A). The proportion of CD99^+^ cells was significantly higher in T-ALL (median 90.5%, range 27.4–97%) than BM MNC (22%, 6.8–51.9%), HSC (22%, 10–62.7%) and CD3^+^ T cells (0.55%, 0.14–15.9%, P≤0.004). In normal donor samples, the proportion of CD99^+^ cells was significantly lower in CD3^+^ T cells compared to HSC and MNC (P≤0.007). Subsequently, we investigated expression of CD99 in known LPC subpopulations[5, 6] from these T-ALL cases. The highest proportions of CD99^+^ cells were found in the CD34^+^/CD7^+^ (median 91.7%, range 52–99.6%) and CD34^-^/CD7^+^ LPC populations (97.3, 40–99.3%, Fig 1B). Significantly fewer CD99^+^ cells were detected in the CD34^+^/CD7^-^ and CD34^-^/CD7^-^ subpopulations with median values of 29.9 and 21.7%, respectively (P≤0.009). However, with the exception of pts 2 and 8, the proportions of CD99^+^ cells detectable in the CD34^+^/CD7^-^ and CD34^-^/CD7^-^ subpopulations were above 20%. In order to compare individual patient samples, the proportions of CD99^+^ cells in each LPC subpopulation are also shown (Fig 1C). Whilst there are clear differences between cases, in general the proportion of CD99^+^ cells in the CD34^+^/CD7^+^ subpopulation was similar across the cohort. Likewise, expression of CD99 was similar in the CD34^-^/CD7^+^ subpopulation, with the exception of pts 3 and 6. To further investigate this CD99 overexpression WGA analyses were performed on sorted subpopulations from 5 cases, based on expression of CD34 and CD7 and unsorted bulk leukemia cells. Results were compared with data from normal BM MNC and CD34^+^/CD38^-^ HSC, (www.ebi.ac.uk/arrayexpress, accession number E-MTAB-4006). CD99 was expressed at higher levels in ALL cells and some LPC subpopulations compared to BM MNC and HSC (Fig 1D), although the differences were not significant. Other genes associated with T-ALL, such as CD2 and CD5, were 2 and 7-fold overexpressed, respectively, compared to normal BM MNC.

**Fig 2:**
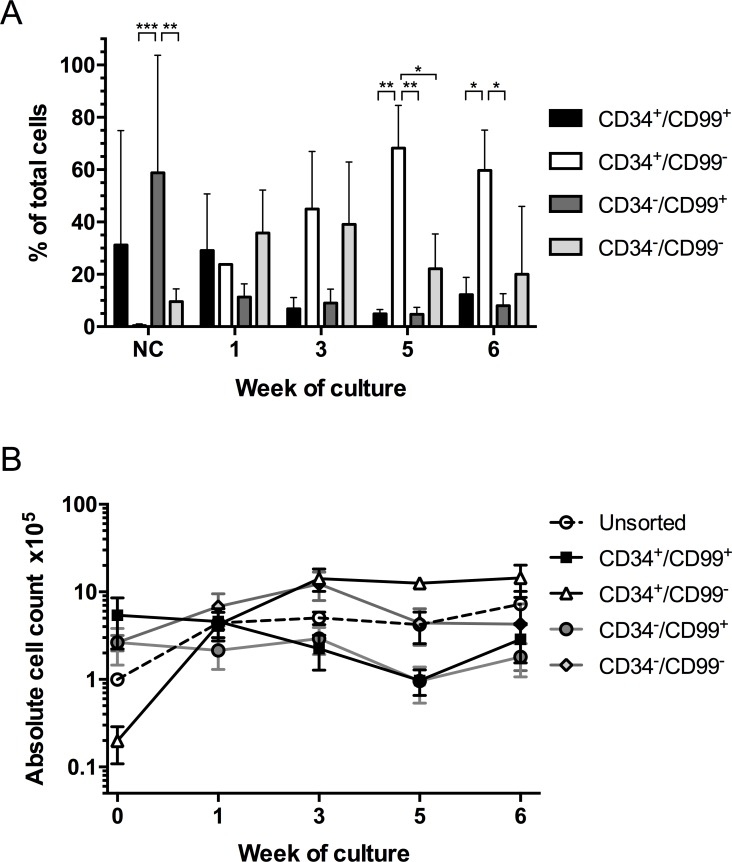
In vitro proliferation of CD34/CD99 subpopulations. (A) T-ALL cells (pts 1, 4–6 and 9) were stained with antibodies against CD34, CD99 and CD7, the subpopulations were sorted based on CD34/CD99 expression and the proliferative ability was assessed in long-term culture. Cultures were maintained with weekly half-media changes. Absolute cell counts, derived from each sorted population and unsorted controls, were determined by flow cytometry at weeks 1, 3, 5 and 6. (A) Proportions of total viable cells, represented by each sorted population, were calculated using absolute cell count values at the time points indicated. Data expressed as mean ± SD, * P = 0.02, ** P≤0.005, *** P = 0.0004. (B) Maintenance and expansion of unsorted cells and each sorted subpopulation over the time course of the culture. NC, nucleated cells.

As the FCM analyses demonstrated CD99 was expressed on all subpopulations that we have previously shown to have LPC capacity, we investigated whether its expression was important for proliferation in vitro and in vivo. Since CD99 expression co-segregated with CD7 in most cases, CD34 and CD99 subpopulations were purified for functional analyses. On sorting, the CD34^+^/CD99^+^ and CD34^-^/CD99^+^ subpopulations represented the majority of leukemia blasts (31.5±43% and 62.2±44%, respectively), while the CD34^+^/CD99^-^ subpopulation was the smallest (0.40±0.7%, Fig 2A). After 3 weeks in culture, the majority of proliferating cells were derived from the CD34^+^/CD99^-^ and CD34^-^/CD99^-^ subpopulations. Cells derived from the CD34^+^/CD99^-^ subpopulation accounted for the majority of proliferating cells at weeks 5 (68.2±16%) and 6 (60.0±15%, P = 0.02). Greatest expansion was observed in cultures of CD34^+^/CD99^-^ cells (4.6–1798 fold, *P*≤0.02) from 7.5x10^3^ up to 2.6x10^6^ cells at week 6 (Fig 2B). Expansion was also observed in cultures of unsorted cells from 1x10^5^ to >7x10^5^ and CD34^-^/CD99^-^ cells (4.7 fold at week 3, 2.5 fold at week 6). In cultures of CD99^+^ cells, absolute cell counts initially declined but recovered to 57% (CD34^+^/CD99^+^ subpopulation) or 70% (CD34^-^/CD99^+^ subpopulation) of initial starting cell numbers by week 6. FISH analyses confirmed the cultured cells from each sorted subpopulation retained the patient specific karyotypic abnormalities and the frequency of aberrant cells (20–100% FISH^+^) was similar to that reported at diagnosis, confirming proliferation of cells derived from the leukemia clone.

Cells from 5 cases were sorted based on expression of CD34 and CD99 (detailed in Table 1) and inoculated into NSG mice at a range of doses to estimate the frequency of LPC (Table 2). LPC were detected in each subpopulation examined. The frequencies varied from 1 in 1300 to 1 in 7.4x10^4^ but were highest in the CD34^+^/CD99^-^ subpopulation. As the number of sorted cells available limited the extent of the dilution analysis, these LPC frequencies are likely to be an underestimate.

**Table 2:** Frequency of LPC in CD34/CD99 subpopulations.

Population	Dose	Sample ID	No. engrafted / No. injected	LPC frequency (95% CI)	% FISH ^+^
**Unsorted**	5x10^6^	2	1/1	1: 1.3x10^5^ (0.4x10^5^–3.8x10^5^)	72–100
	4x10^6^	6,9	3/3		
	2x10^6^	4,9	3/3		
	1x10^6^	2,4,9	4/4		
	5x10^5^	3,6,9	3/3		
	1x10^5^	3,9	1/2		
	5x10^4^	2,4,9	1/3		
	1x10^4^	3	0/1		
**CD34^+^/CD99^+^**	4x10^6^	6	1/1	1: 3.3x10^4^ (0.1x10^5^–1.1x10^5^)	66–100
	2x10^6^	4	1/1		
	1.5x10^5^	9	1/1		
	1x10^5^	4,6	3/4		
	3x10^4^	2	1/1		
	1x10^4^	2	1/1		
	3x10^3^	3	1/1		
**CD34^+^/CD99^-^**	1x10^5^	9	1/1	1: 1.3x10^3^ (0.3x10^3^–5.6x10^3^)	75–100
	6x10^4^	4	1/1		
	5x10^4^	2,4	2/2		
	3x10^4^	4	1/1		
	1x10^4^	3	1/1		
	2.3x10^3^	3,6	2/2		
	1x10^3^	6	0/1		
**CD34^-^/CD99^+^**	2.5x10^6^	4	1/1	1: 5.6x10^4^ (0.2x10^5^–1.6x10^5^)	70–100
	2.2x10^6^	9	1/1		
	1.6x10^6^	2	1/1		
	1x10^6^	4,9	3/3		
	5x10^5^	2	1/1		
	4x10^5^	3	1/1		
	3x10^5^	6	1/1		
	1x10^5^	3,4,6,9	3/4		
	5x10^4^	2	1/1		
	2x10^6^	2	1/1	1: 7.4x10^4^ (0.3x10^5^–1.6x10^5^)	81–100
	1x10^6^	4,9	2/2		
	6x10^5^	4	1/1		
**CD34^-^/CD99^-^**	2x10^5^	3	1/1		
	1x10^5^	2,3,4,6,9	5/7		
	5x10^4^	2,3	1/2		

LPC frequencies calculated using L-Calc Software

**Fig 3:**
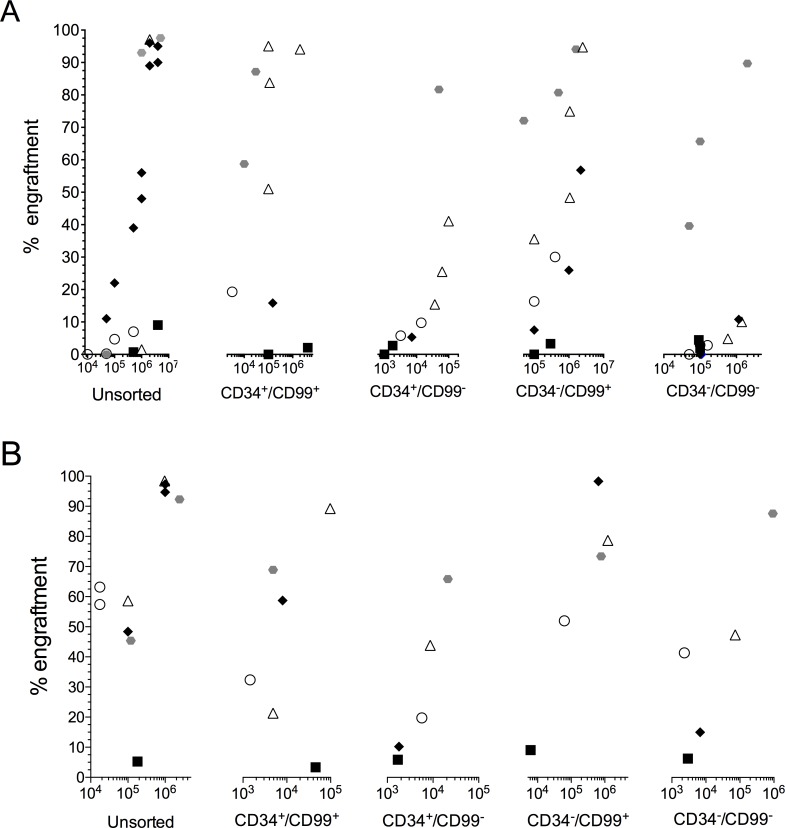
NSG engrafting capacity of CD34/CD99 subpopulations. T-ALL cells from 5 cases (pts 2, 3, 4, 6, 9) were sorted based on expression of CD34/CD99 and the repopulating capacity of each sorted population was assessed in NSG mice (A). Each patient is represented by a specific symbol and each symbol depicts the engraftment detected in the BM of an individual mouse by flow cytometric analysis, measured by anti-human CD45 antibody. (B) Cells harvested from the BM of some engrafted primary recipients were inoculated into secondary NSG recipients. The X axes depict the number of cells inoculated from each sorted subpopulation (A) or from primary xenograft source (B).

Leukemia engraftment in NSG mice was achieved using unsorted cells (0.7–98%) and with each sorted subpopulation, CD34^+^/CD99^+^ (2–99%), CD34^+^/CD99^-^ (3–85%), CD34^-^/CD99^+^ (3–99%) and CD34^-^/CD99^-^ (2–93%, Fig 3A). In most cases there was no difference in the rate of engraftment between the different subpopulations, with engraftment achieved 3–12 weeks from inoculation. This was despite injecting 2–3 log fewer CD99^-^ cells. In pt 4, engraftment of both CD34^+^/CD99^-^ and CD34^-^/CD99^-^ cells was delayed by 3 weeks compared to recipients of CD34^+^/CD99^+^ and CD34^-^/CD99^+^ cells. However, up to 2 log fewer CD99^-^ cells were inoculated. All 4 subpopulations also had self-renewal capacity as demonstrated by engraftment of secondary NSG recipients (Fig 3B). Higher levels of leukemia engraftment were observed in the secondary animals compared to the primary recipients, despite injecting lower numbers of cells in some cases (*P*≤0.04). Time to leukemia onset was reduced compared to the primary animals (2–9 weeks) and in pt 4, the engraftment delay between CD99^+^ and CD99^-^ subpopulations was reduced to 12 days. In most cases, regardless of the subpopulation inoculated, the immunophenotype of harvested NSG BM cells was similar to that of the patients at diagnosis (Table A in S1 File)), with loss of CD34 and gain of CD99 in pts 2, 3 and 9 and gain of both markers in pt 6 (Table B in S1 File). In pt 4 there were no substantive changes in immunophenotype with passage through NSG mice, reflecting the mixed expression of these antigens in the patient at diagnosis. Patient specific TCR rearrangements were conserved with passage through NSG mice (Table C in S1 File) and FISH analyses showed the cells also had the patient specific karyotypic aberrations (66–100%), confirming engraftment of leukemia cells and demonstrating each subpopulation had self-renewal capacity.

## Discussion

While modern treatment protocols have vastly improved survival rates for childhood leukemia, the 5 year survival rate for T-ALL (~85%) remains worse than that of BCP-ALL.[23] The majority of relapses occur during treatment and the prognosis for these patients is poor, with <25% surviving.[23, 24] A better understanding of the biology of the disease may permit the development of more targeted, less toxic therapies. Over-expression of certain antigens on ALL cells enables accurate diagnosis, classification, disease monitoring and importantly, indicates potential targets for therapy. While several antibody based therapies have been developed for BCP-ALL, options for T-ALL therapy are limited. Lack of specificity of some markers and clonal heterogeneity within patient cells are important considerations in the development of targeted therapeutics. In the present study we sought to investigate the relevance of CD99 over-expression in childhood T-ALL.

The data from FCM and genetic analyses confirmed CD99 expression is higher in T-ALL cases than in normal BM cells.[16, 17] In addition, the FCM findings are consistent with previous reports, using the same monoclonal antibody clone, identifying CD99 as a useful discriminator of T-ALL blasts from normal T lymphocytes.[15, 16] During T cell development CD99 is downregulated on normal T-cells with increasing expression of surface CD3.[25] CD99 was highly expressed in the current patient cohort, regardless of prognostic indicators or MRD risk status. One of the 9 cases studied, pt 3, a relapse sample classed as high MRD risk, had comparatively low expression of CD99 (27%). This is also consistent with reported frequencies of lower CD99 expression in ~15% of childhood T-ALL cases.[16] While CD99 gene expression was not significantly higher, in a smaller cohort of patients, compared to normal BM samples, genes for other leukemia associated phenotype markers used in the EuroFlow FCM T-ALL MRD panels, including CD2 and CD5, were overexpressed in these cases.[15] Gene expression analyses of LPC are extremely challenging, due to limiting cell numbers and this study represents the first report on WGA analyses in T-ALL LPC populations.

In order to understand the relevance of this overexpression of CD99 and to determine whether it plays a role in leukemogenesis, it was essential to assess the functional capacity of these cells in vitro and in vivo. We have previously shown that blasts from both BCP- and T-ALL cases can be maintained in culture for extended periods of time with no loss of karyotype or TCR rearrangements.[2, 5] Data from the long-term suspension cultures and serial NSG passage indicate that expression of CD99 is not a prerequisite for proliferation in vitro or for leukemia propagating capacity in vivo. While the levels of engraftment varied amongst patient samples and the subpopulations inoculated, engraftment was achieved in every case with both CD99^+^ and CD99^-^ cells, even though the majority of blast cells in these cases expressed CD99. Likewise, both CD34^+^ and CD34^-^ cells could engraft, regardless of expression of CD99, despite 3 of the 5 patients having a CD34^-^ immunophenotype. The lower levels of engraftment observed with CD99^-^ cells is likely to be a result of lower numbers of these cells in the inocula, rather than reduced engrafting capacity. In fact, the analyses of LPC frequency indicate it was higher in the CD34^+^/CD99^-^ subpopulation in this patient cohort. In each sample studied, the phenotype of the inoculated cells changed in vivo to produce T-ALL in NSG mice with an immunophenotype similar to that of the patient at diagnosis, with increased expression of CD99 in recipients of CD99^-^ subpopulations. Such immunophenotypic changes following xenotransplantion of T-ALL cells are consistent with previous reports using NSG and NOD/SCID mice.[2, 5] All the sorted subpopulations had self-renewal capacity and engraftment was higher and leukaemia onset more rapid in the secondary animals. Furthermore, the patient specific karyotypes and TCR gene rearrangements were also retained with passage through NSG mice, confirming engraftment of leukemia cells. These data suggest that targeting CD99 alone, or in combination with CD34/CD7, would not be an effective approach to eradicating cells with leukaemia initiating capacity in vivo.

In addition, the findings indicate that absence of CD99^+^ cells during therapy may not mean lack of residual disease. However, the established practice of multi-panel FCM MRD analyses should mitigate the risk of false negative results. A previous study reported loss of both CD99 and TdT during therapy.[17] The authors suggested this could be due to selective survival of CD99^-^ subpopulations or downregulation of CD99 on surviving cells. While our study did not investigate antigenic changes during therapy, we did not observe loss of CD99 during passage in NSG. As our data demonstrate that both CD99^+^ and CD99^-^ subpopulations were capable of self-renewal, survival of only CD99^-^ cells following induction therapy, as suggested by Roshal et al, [17] would still leave a population of cells potentially capable of causing relapse. Such a phenomenon has been reported in patients with BCP ALL relapsing with CD19^-^ leukemias after administration of CD19-CAR-T cells, with lymphoid to myeloid phenotypic switching in some cases.[21, 22, 26]. The current findings add to the evidence of the diverse nature of this malignancy and are further proof that multiple LPC subpopulations exist in childhood T-ALL, as has been described for BCP ALL.[5, 27, 28] In order to improve outcomes for T-ALL it will be necessary to target all cells that have leukemia propagating capacity in vivo and not just those that represent the majority of blast cells in patients.

## Supporting Information

S1 File Supporting Tables and Figure. (DOCX)
