# Supplementary material for: Investigating CD99 Expression in Leukemia Propagating Cells in Childhood T Cell Acute Lymphoblastic Leukemia
Source: PLoS One. 2016 Oct 20;11(10):e0165210. doi: 10.1371/journal.pone.0165210 (PMC5072597; doi:10.1371/journal.pone.0165210)
Supplement: S1 File — (DOCX) [file pone.0165210.s001.docx]

**Supporting Tables and Figure**

**Table A Clinical details of T-ALL cases studied**

| **ID** | **Karyotype** | **Age (y)** | **Sex** | **Disease**  **status** | **Risk status** | **Blast infiltration of BM (%)** | **Immunophenotype at diagnosis** |
| --- | --- | --- | --- | --- | --- | --- | --- |
| 1 | iso 9, +4 | 15 | M | Relapse | Risk | >90 | CD2+, cCD3+, CD7+, CD99+, TdT^+^ |
| 2 | t(11;14) | 2 | M | Relapse | Low | >90 | CD2+, cCD3+, CD5+, CD4+, CD7+, CD8+, CD99+ |
| 3 | t(9;16),+7, -9 | 5 | M | Relapse | Risk | >95 | CD2+, cCD3+, CD5+, CD7^weak^, CD8+, CD99+ |
| 4 | 46 XY | 17 | M | Diagnosis | Risk | >95 | CD1a+, CD2+, cCD3+, CD4+, CD5+, CD7+, CD8+, CD34+, CD99+, TdT+ |
| 5 | t(1;14) | 10 | M | Diagnosis | Low | >90 | CD2+, cCD3+, CD5+, CD7+, CD13+, CD99+, TdT^weak^ |
| 6 | del 6q | 15 | M | Diagnosis | Risk | >90 | CD1a^weak^, CD2+, cCD3+, CD5+, CD7+, CD13, CD34+, CD99+, TdT+ |
| 7 | +1, del 6, del11 | 2 | F | Diagnosis | N/A | >90 | CD2+, CD7+, CD5+, cCD3+, CD99+, TCRαβ^+^, TdT- |
| 8 | 46 XY | 3 | M | Diagnosis | Risk | >95 | CD2+, cCD3+, CD7+, CD5+, CD4+, CD8+, CD99+, TCRαβ-, TCRγδ-, TdT^weak^ |
| 9 | complex | 6 | M | Diagnosis | Low | 87 | CD2+, cCD3+, sCD3-, CD7+, CD99+, TdT+ |

**Table B Immunophenotypic and karyotypic analyses of NSG BM**

| **Patient** | **Subfraction** | **Immunophenotype *** | | | **% FISH ^+^** |
| --- | --- | --- | --- | --- | --- |
|  | **Inoculated** | **%CD7^+^** | **%CD34^+^** | **%CD99^+^** |  |
| 2 | Unsorted | 75.82 | 3.03 | 95.54 | 100 |
|  | CD34^+^/CD99^+^ | 89.15 | 4.00 | 89.11 | 100 |
|  | CD34^+^/CD99^-^ | 84.37 | 2.0 | 83.60 | 100 |
|  | CD34^-^/CD99^+^ | 96.24 | 0.06 | 96.47 | 100 |
|  | CD34^-^/CD99^-^ | 95.22 | 0 | 93.53 | 100 |
|  |  |  |  |  |  |
| 3 | Unsorted | 29.78 | 4.74 | 68.04 | 80 |
|  | CD34^+^/CD99^+^ | 18.43 | 6.12 | 53.80 | 84 |
|  | CD34^+^/CD99^-^ | 24.49 | 2.42 | 65.40 | 75 |
|  | CD34^-^/CD99^+^ | 14.02 | 0.06 | 44.06 | 70 |
|  | CD34^-^/CD99^-^ | 9.42 | 0.08 | 39.10 | - |
|  |  |  |  |  |  |
| 4 | Unsorted | 92.27 | 71.90 | 98.67 | - |
|  | CD34^+^/CD99^+^ | 93.76 | 73.20 | 98.39 | - |
|  | CD34^+^/CD99^-^ | 13.26 | 46.70 | 26.03 | - |
|  | CD34^-^/CD99^+^ | 86.75 | 9.02 | 91.24 | - |
|  | CD34^-^/CD99^-^ | 24.27 | 3.11 | 14.92 | - |
|  |  |  |  |  |  |
| 6 | Unsorted | 89.00 | 62.38 | 72.70 | 72 |
|  | CD34^+^/CD99^+^ | 72.13 | 74.44 | 88.10 | - |
|  | CD34^+^/CD99^-^ | 65.17 | 58.41 | 70.70 | - |
|  | CD34^-^/CD99^+^ | 83.9 | 54.20 | 96.50 | 71 |
|  | CD34^-^/CD99^-^ | 83.2 | 61.47 | 62.02 | 85 |
|  |  |  |  |  |  |
| 9 | Unsorted | 99.93 | 2.1 | 89.33 | 91 |
|  | CD34^+^/CD99^+^ | 86.46 | 3.4 | 74.27 | 66 |
|  | CD34^+^/CD99^-^ | 53.85 | 0.17 | 55.09 | 80 |
|  | CD34^-^/CD99^+^ | 99.08 | 1.15 | 86.49 | 100 |
|  | CD34^+^/CD99^-^ | 59.13 | 0 | 63.82 | 81 |

* Immunophenotype of human CD45^+^ cells removed from murine BM. All samples analyzed were negative for CD19.

**Table C Details of secondary NSG transplants**

| **Patient** | **1^o^ NSG source** | **Total CD45^+^ cells inoculated (x10^4^)** | **No. CD99^+^ cells inoculated (x10^4^)*** | **% Engraftment** |
| --- | --- | --- | --- | --- |
| 2 | Unsorted | 12-240 | 11.5-233 | 46-92 |
|  | CD34^+^/CD99^+^ | 0.48 | 0.43 | 69 |
|  | CD34^+^/CD99^-^ | 2.12 | 1.77 | 66 |
|  | CD34^-^/CD99^+^ | 78.3 | 75.6 | 73 |
|  | CD34^-^/CD99^-^ | 92.9 | 86.9 | 87 |
|  |  |  |  |  |
| 3 | Unsorted | 1.75 | 1.19 | 63 |
|  | CD34^+^/CD99^+^ | 0.15 | 0.08 | 32 |
|  | CD34^+^/CD99^-^ | 0.57 | 0.37 | 20 |
|  | CD34^-^/CD99^+^ | 6.25 | 2.76 | 52 |
|  | CD34^-^/CD99^-^ | 0.23 | 0.09 | 41 |
|  |  |  |  |  |
| 4 | Unsorted | 10-97 | 9.87-95 | 58-98 |
|  | CD34^+^/CD99^+^ | 0.48-9.77 | 0.47-9.61 | 89 |
|  | CD34^+^/CD99^-^ | 0.87 | 0.22 | 44 |
|  | CD34^-^/CD99^+^ | 123 | 112 | 78 |
|  | CD34^-^/CD99^-^ | 7.28 | 1.09 | 47 |
|  |  |  |  |  |
| 6 | Unsorted | 18.1 | 13.2 | 5 |
|  | CD34^+^/CD99^+^ | 4.56 | 4.01 | 3 |
|  | CD34^+^/CD99^-^ | 0.17 | 0.12 | 6 |
|  | CD34^-^/CD99^+^ | 0.62 | 0.60 | 9 |
|  | CD34^-^/CD99^-^ | 0.29 | 0.18 | 6 |
|  |  |  |  |  |
| 9 | Unsorted | 10-100 | 8.93-89.3 | 48-97 |
|  | CD34^+^/CD99^+^ | 0.80 | 0.59 | 59 |
|  | CD34^+^/CD99^-^ | 0.18 | 0.09 | 10 |
|  | CD34^-^/CD99^+^ | 65.0 | 56.3 | 98 |
|  | CD34^+^/CD99^-^ | 0.68 | 0.43 | 15 |
|  |  |  |  |  |

* Number CD99^+^ cells inoculated calculated based on immunophenotype of cells recovered from BM of primary NSG mice (detailed in Supplementary Table 2).

**Table D Sequence analyses of TCR rearrangements in cells from engrafted NSG mice**

| **Sample source** | | **Clonal rearrangement** | | | | | | | | | | |  |
| --- | --- | --- | --- | --- | --- | --- | --- | --- | --- | --- | --- | --- | --- |
|  | |  |  | | |  | | |  | | |  |  |
| Pt. 2 | |  |  | | |  | | |  | | |  |  |
| Unsorted NSG 1° | | (TRGV2*01) | tgtgccacc......... | | *aaatccccctcatgg* | | | ............gaaactcttt | | | (TRGJ1*01) | | |
|  | |  |  | |  | | |  | | |  | | |
| CD34^+^/CD99^+^ NSG 1° | | (TRGV9*01) | tgtgccacc......... | | *aaatccccctcatgg* | | | ............gaaactcttt | | | (TRGJ1*01) | | |
|  | |  |  | |  | | |  | | |  | | |
| CD34^+^/CD99^-^ NSG 1° | | (TRGV9*01) | tgtgccacc......... | | *aaatccccctcatgg* | | | ............gaaactcttt | | | (TRGJ1*01) | | |
|  | |  |  | |  | | |  | | |  | | |
| CD34^-^/CD99^+^ NSG 1° | | (TRGV9*01) | tgtgccacc......... | | *aaatccccctcatgg* | | | ............gaaactcttt | | | (TRGJ1*01) | | |
|  | |  |  | |  | | |  | | |  | | |
| CD34^-^/CD99^-^ NSG 1° | | (TRGV9*01) | tgtgccacc......... | | *aaatccccctcatgg* | | | ............gaaactcttt | | | (TRGJ1*01) | | |
|  | |  |  | |  | | |  | | |  | | |
| Pt. 4 | |  |  | |  | | |  | | |  | | |
| Unsorted NSG 1° | | (TRGV9*01) | tgtgcc............ | | *ccgggagg* | | | ...ttattataagaaactcttt | | | (TRGJ1*01) | | |
| Unsorted NSG 2° | | (TRGV9*01) | tgtgcc............ | | *ccgggagg* | | | ...ttattataagaaactcttt | | | (TRGJ1*01) | | |
|  | |  |  | |  | | |  | | |  | | |
| CD34^+^/CD99^+^ NSG 1° | | (TRGV9*01) | tgtgcc............ | | *ccgggagg* | | | ...ttattataagaaactcttt | | | (TRGJ1*01) | | |
| CD34^+^/CD99^+^ NSG 2° | | (TRGV9*01) | tgtgcc............ | | *ccgggagg* | | | ...ttattataagaaactcttt | | | (TRGJ1*01) | | |
|  | |  |  | |  | | |  | | |  | | |
| CD34^+^/CD99^-^ NSG 1 | | (TRGV9*01) | tgtgcc............ | | *ccgggagg* | | | ...ttattataagaaactcttt | | | (TRGJ1*01) | | |
| CD34^+^/CD99^-^ NSG 2° | | (TRGV9*01) | tgtgcc............ | | *ccgggagg* | | | ...ttattataagaaactcttt | | | (TRGJ1*01) | | |
|  | |  |  | |  | | |  | | |  | | |
| CD34^-^/CD99^+^ NSG 1° | | (TRGV9*01) | tgtgcc............ | | *ccgggagg* | | | ...ttattataagaaactcttt | | | (TRGJ1*01) | | |
| CD34^-^/CD99^+^ NSG 2° | | (TRGV9*01) | tgtgcc............ | | *ccgggagg* | | | ...ttattataagaaactcttt | | | (TRGJ1*01) | | |
|  | |  |  | |  | | |  | | |  | | |
| CD34^-^/CD99^-^ NSG 1° | | (TRGV9*01) | tgtgcc............ | | *ccgggagg* | | | ...ttattataagaaactcttt | | | (TRGJ1*01) | | |
| CD34^-^/CD99^-^ NSG 2° | | (TRGV9*01) | tgtgcc............ | | *ccgggagg* | | | ...ttattataagaaactcttt | | | (TRGJ1*01) | | |
|  |  | | |  | | |  | | |  | | |  |

Unique V and J segments are show in parentheses. Randomly deleted bases are represented by full stops. Randomly inserted bases (both N and P nucleotides) are in italics, 1° and 2° indicate primary and secondary NSG mice.


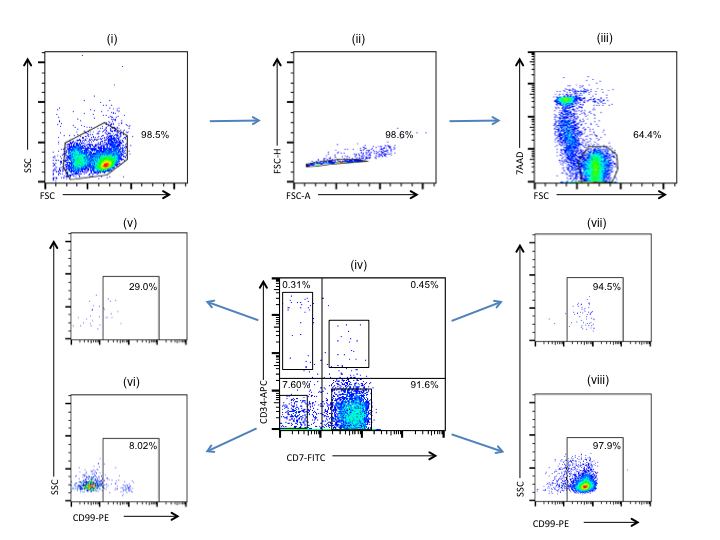


**Figure A Gating strategy for flow cytometric analyses and sorting**

T-ALL blast cells were initially gated on the basis of low forward and side scatter (i), doublets were excluded using a FSC area (integral) vs height (peak) plot (ii), then live 7AAD negative cells selected (iii). Subsequently cells were gated for expression of CD34-APC and CD7-FITC (iv). Sort gates were established using FMO controls and were separated by at least 10 channels, as shown. The proportion of CD99-PE^+^ cells in each of the sorted population is shown (v-viii). A similar strategy was used to sort CD34/CD99 subpopulations with subsequent analyses of CD7 expression in each subpopulation.
